# Barriers to Genetic Testing in Vascular Malformations

**DOI:** 10.1001/jamanetworkopen.2023.14829

**Published:** 2023-05-23

**Authors:** Alexandra J. Borst, Adrienne M. Hammill, Shelley E. Crary, Thomas W. McLean, Thomas Felton, Julie Blatt

**Affiliations:** 1 Division of Hematology, The Children’s Hospital of Philadelphia, Philadelphia, Pennsylvania; 2 Division of Oncology, The Children’s Hospital of Philadelphia, Philadelphia, Pennsylvania; 3 Division of Hematology, Cancer and Blood Diseases Institute, Cincinnati Children’s Hospital Medical Center, Cincinnati, Ohio; 4 Department of Pediatrics, University of Cincinnati College of Medicine, Cincinnati, Ohio; 5 Division of Pediatric Hematology-Oncology, University of Arkansas for Medical Sciences and Arkansas Children’s Hospital, Little Rock; 6 Section of Pediatric Hematology-Oncology, Atrium Health Wake Forest Baptist, Winston-Salem, North Carolina; 7 McLendon Clinical Laboratories, UNC Health, The University of North Carolina, Chapel Hill; 8 Division of Pediatric Hematology-Oncology, The University of North Carolina, Chapel Hill

## Abstract

**Importance:**

Vascular malformations (VMs) are rare disorders of vasculogenesis associated with substantial morbidity. Improved understanding of their genetic basis is increasingly guiding management, but logistical barriers to obtaining genetic testing in patients with VM may constrain treatment options.

**Objectives:**

To examine the institutional mechanisms for and obstacles to obtaining genetic testing for VM.

**Design, Setting, and Participants:**

This survey study invited members of the Pediatric Hematology-Oncology Vascular Anomalies Interest Group, representing 81 vascular anomaly centers (VACs) serving individuals up to 18 years of age, to complete an electronic survey. Respondents were mostly pediatric hematologists-oncologists (PHOs) but included geneticists, genetic counselors, clinic administrators, and nurse practitioners. Responses that were received between March 1 and September 30, 2022, were analyzed with descriptive methods. Requirements for genetic testing by several genetics laboratories were also reviewed. Results were stratified by size of the VAC.

**Main Outcomes and Measures:**

Vascular anomaly center and associated clinician characteristics and practice patterns for ordering and obtaining insurance approval for genetic testing for VMs were collected.

**Results:**

Responses were received from 55 of 81 clinicians, for a response rate of 67.9%. Most respondents were PHOs (50 [90.9%]). Most respondents (32 of 55 respondents [58.2%]) replied that they order genetic testing on 5 to 50 patients per year and reported a genetic testing volume increase of 2- to 10-fold over the past 3 years (38 of 53 respondents [71.7%]). Most testing was ordered by PHOs (35 of 53 respondents [66.0%]), followed by geneticists (28 [52.8%]) and genetic counselors (24 [45.3%]). In-house clinical testing was more common at large and medium-sized VACs. Small VACs were more likely to use oncology-based platforms, which potentially miss low-frequency allelic variants in VM. Logistics and barriers varied by size of the VAC. Obtaining prior authorization was the duty shared among PHOs, nurses, and administrative staff, but the burden of insurance denials and appeals were on PHOs (35 of 53 respondents [66.0%]). Lack of administrative support; unclear institutional, insurance, and laboratory requirements; and lack of clinician education were barriers to genetic testing at VACs of all sizes. The effort to obtain genetic testing for patients with VM, compared with patients with cancer, was perceived as excessive, despite genetic testing being considered standard of care for this population.

**Conclusions and Relevance:**

Results of this survey study showed the barriers to genetic testing for VM across VACs, described differences between VACs based on size, and proposed multiple interventions to support clinicians ordering genetic testing for VM. The results and recommendations should have broader application to clinicians caring for patients for whom molecular diagnosis is important to medical management.

## Key Points

**Question:**

What are the barriers to obtaining genetic information for patients with vascular malformation (VM)?

**Findings:**

In this survey study of 55 vascular anomaly specialists (primarily pediatric hematologists-oncologists), barriers to genetic testing for VM were identified across vascular anomaly centers of all sizes, including lack of administrative support; unclear institutional, insurance, and laboratory requirements; and lack of clinician education.

**Meaning:**

Findings from this survey suggest that, despite being critical to the comprehensive care of patients with VM, obtaining genetic information is difficult; study results and recommendations should have broader application to clinicians caring for patients for whom molecular diagnosis is important to medical management.

## Teaser

This survey study reports on the current logistics and challenges of ordering and coordinating insurance authorization for genetic testing for pediatric patients with vascular anomalies.

## Introduction

Vascular malformations (VMs) are a heterogeneous group of disorders that arise from disrupted vascular morphogenesis and are primarily driven by somatic variants in genes of the PI3K/AKT/mTOR (phosphoinositide 3-kinase/protein kinase B/mammalian target of rapamycin) and Ras/MAPK (mitogen-activated protein kinase) angiogenesis signaling pathways.^1,2,3,4,5,6^ Lesion-based genetic testing of VMs and comparison with genetic testing on blood is often needed for molecular characterization.^7,8^ Over the past decade, the identification of driver sequence variations in VMs has led to interest in targeted therapies, many adopted from oncology.^1,9,10^ Molecular information is becoming crucial to optimal management of patients with VMs; thus, sensitive and accurate genetic testing is now a key component of comprehensive care.

In practice, obtaining genetic studies is complicated and requires determination of insurance coverage in addition to sample acquisition. Procedures are often performed on children, with concerns about painful diagnostic procedures and anesthetic risks. Compared with cancers, most VMs harbor somatic pathogenic variants at lower variant allele frequencies (often <5% to 10%), requiring sensitive sequencing methods.^2,11,12^ Currently, few laboratories in the US offer testing specifically for this patient population.

To examine the institutional mechanisms for and obstacles to obtaining genetic testing for VM, we surveyed members of the Pediatric Hematology-Oncology Vascular Anomalies (PHO VA) Interest Group and reviewed requirements for genetic testing from several laboratories and insurance companies. The results identified gaps in the ability to optimize care for patients with VM. Herein, we offered guidance for successful genetic testing while minimizing clinician and institutional effort.

## Methods

### Survey

In March 2022, we sent an electronic survey using REDCap, version 13.4.11 (Vanderbilt University) to members of the PHO VA Interest Group, including primarily pediatric hematologists-oncologists (PHOs) representing 81 vascular anomaly centers (VACs) across the US. The Children’s Hospital of Philadelphia deemed this study exempt from institutional review board approval and waived the informed consent requirement because the study collected no patient data or protected health information and involved only survey procedures. We followed the American Association for Public Opinion Research (AAPOR) reporting guideline.

The survey (eAppendix in Supplement 1) asked about VAC size, VAC composition, logistics of ordering genetic testing, and issues with insurance authorization.^13^ Respondent race and ethnicity, sex, and gender identity data were not collected. Vascular anomaly center size was categorized as small (<25 unique patients with VM per year), medium (25-100 patients per year), or large (>100 patients per year). The survey focused on patients with somatic postzygotic disorders causing VM, but some respondents included notes about testing for germline conditions. Because most respondents also cared for pediatric patients with cancer, they were asked to subjectively compare the ease of obtaining genetic studies for patients with VM vs patients with cancer.

After 4 weeks, nonrespondents were sent an individualized follow-up email, and nonresponse triggered contact with another member from that VAC. Responses received between March 1 and September 30, 2022, were included in this analysis.

### Billing Procedures for Genetics Laboratories

The websites of the genetics laboratories that were most commonly used by respondents (Invitae, University of Pennsylvania Genetic Diagnostic Laboratory, Seattle Children’s Hospital Molecular Genetics Laboratory [which was mistakenly referred to as University of Washington laboratory in the survey], and Washington University in St Louis NGS Laboratory) were reviewed for information on ordering, preauthorization, and billing requirements. Managers in these laboratories were contacted for clarification, if needed. An effort was made to determine the demographic and disease-specific information required from the originating clinic, whether preauthorization was needed, and whether responses varied with patient insurance coverage.

### Statistical Analysis

Data were stratified by VAC size. Data were analyzed using descriptive methods.

## Results

### Characteristics of Survey Respondents and VACs

There were 55 survey respondents from 81 requests (67.9% response rate). Most respondents were PHOs (50 of 55 respondents [90.9%]); other respondents included geneticists, genetic counselors, clinic administrators, and nurse practitioners involved in the care of patients with VM (Table 1). Of the 55 respondents, approximately half (27 [49.1%]) were from large VACs; 17 (30.9%) reported being from midsize programs, and 11 (20.0%) were from small clinics. Most respondents from large and midsize VACs reported having a dedicated program for vascular anomalies. All large centers and 60% of the midsize centers had either a structured multidisciplinary clinic or a dedicated team of physicians from different specialties. Most of the small centers (7 of 11 [63.6%]) had no structured team or conference. All VACs cared for infants, children, and adolescents up to 18 years of age. Twenty-nine centers of the 55 centers (52.7%) followed children and adults of all ages, although in most cases vascular anomalies–specific medical care was provided by pediatric rather than adult specialists.

**Table 1.** Characteristics of 55 Survey Respondents and Associated VAC

Characteristic	Respondents, No. (%)		
	Large VAC (n = 27)	Midsize VAC (n = 17)	Small VAC (n = 11)
Type of institution			
Public	14 (51.9)	11 (64.7)	6 (54.5)
Private	13 (48.1)	6 (35.3)	5 (45.5)
Clinic or program for vascular anomalies			
Yes	24 (88.9)	11 (64.7)	2 (18.2)
No	NA	NA	6 (54.5)
Sort of^a^	3 (11.1)	6 (35.3)	3 (27.3)
Person answering survey			
PHO	24 (88.9)	15 (88.2)	11 (100)
Other physician (otolaryngologist)	1 (3.7)	NA	NA
Nurse practitioner	1 (3.7)	NA	NA
Geneticist	1 (3.7)	1 (5.9)	NA
Genetic counselor	NA	1 (5.9)	NA
PHO VA Interest Group member			
Yes	24 (88.9)	13 (76.5)	11 (100)
No	3 (11.1)	4 (23.5)	NA
CaNVAS member			
Yes	17 (63.0)	2 (11.8)	NA
No	10 (37.0)	15 (88.2)	11 (100)
ISSVA member			
Yes	21 (77.8)	5 (29.4)	3 (27.3)
No	6 (22.2)	12 (70.6)	8 (72.7)
Maximum age of patients seen in the program			
18 y	NA	1 (5.9)	1 (9.1)
21-30 y	9 (33.3)	6 (35.3)	7 (63.6)
No maximum age	16 (59.3)	10 (58.8)	3 (27.3)
Maximum age depends on location of visit and specialty	2 (7.4)	NA	NA

Abbreviations: CaNVAS, Consortium of Investigators of Vascular Anomalies; ISSVA, International Society for the Study of Vascular Anomalies; NA, not applicable; PHO, pediatric hematologist-oncologist; PHO VA, Pediatric Hematology-Oncology Vascular Anomalies Interest Group; VAC, vascular anomaly center.

^a^ A vascular anomalies team or clinicians are available but there is not a specific clinic dedicated to vascular anomalies.

As a measure of institutional commitment to vascular anomalies, respondents were asked about their membership in the national Consortium of Investigators of Vascular Anomalies (CaNVAS) and the International Society for the Study of Vascular Anomalies (ISSVA). Most members of CaNVAS and ISSVA were from large VACs. Geographic distribution of respondents by institution zip code showed a concentration of VACs, regardless of size, on the East Coast, with fewer sites elsewhere.

### Resources for Ordering Genetic Testing

Respondents reported a wide range in the number of patients per year who underwent genetic testing. Of these respondents, less than a quarter (12 of 53 [22.6%]) ordered testing on fewer than 5 patients per year, more than half (32 of 53 [60.4%]) ordered testing on 5 to 50 patients per year, and only a few (5 of 53 [9.4%]) from the large VACs ordered testing on 50 or more patients per year. Programs of all sizes reported an increase in the number of genetic tests. Nearly half of the respondents (26 of 53 [49.1%]) reported that their volume of genetic testing for patients with vascular anomalies had increased by 2- to 5-fold in the past 3 years. Twelve of 53 respondents (22.6%), most of whom were at large centers, reported that testing had increased more than 10-fold. At 6 of 11 small centers (54.5%), genetic testing numbers had stayed about the same. Most respondents (42 of 53 [79.2%]) reported that more than half of testing was done for pediatric patients 18 years or younger.

Respondents reported a wide variation in the type of clinician who placed orders for genetic testing (Table 2). Those who were most frequently responsible were PHOs (35 of 53 [66.0%]), geneticists (28 of 53 [52.8%]), and genetic counselors (24 of 53 [45.3%]). Large VACs had multiple types of personnel ordering genetic testing. No research assistants, nurse practitioners, or administrative staff were entering orders at midsize or small VACs. Respondents from many centers reported that, when genetic testing was ordered at the time of tissue biopsy, the orders were placed by the performing surgeon or proceduralist.

**Table 2.** Resources for Ordering Genetic Testing Reported by 53 Respondents

Survey question	Respondents, No. (%)		
	Large VAC (n = 27)	Midsize VAC (n = 15)	Small VAC (n = 11)
Who places orders for genetic testing?			
Geneticist	18 (66.7)	8 (53.3)	2 (18.2)
Genetic counselor	16 (59.3)	6 (40.0)	2 (18.2)
PHO	18 (66.7)	11 (73.3)	6 (54.5)
Nurse	1 (3.7)	NA	1 (9.1)
Nurse practitioner	7 (25.9)	NA	NA
Research assistant	1 (3.7)	NA	NA
Other administrative staff (program coordinator)	1 (3.7)	NA	NA
Another physician	9 (33.3)	3 (20.0)	2 (18.2)
Who decides what laboratory to send blood testing to?			
Insurance	9 (33.3)	7 (46.7)	3 (27.3)
Person ordering test	17 (63.0)	10 (66.7)	9 (81.8)
Institution	6 (22.2)	1 (6.7)	2 (18.2)
Genetic counselor	10 (37.0)	4 (26.7)	2 (18.2)
Other or combination	9 (33.3)	2 (13.3)	1 (9.1)
Who decides what laboratory to send tissue testing to?			
Insurance	13 (48.1)	6 (40.0)	2 (18.2)
Person ordering test	19 (70.3)	10 (66.7)	7 (63.6)
Institution	4 (14.8)	2 (13.3)	2 (18.2)
Genetic counselor	7 (25.9)	3 (20.0)	2 (18.2)
Other or combination	8 (29.6)	1 (6.7)	1 (9.1)
Who performs biopsy to obtain tissue specimen for genetic testing?			
Dermatologist	20 (74.1)	9 (60.0)	4 (36.4)
Interventional radiologist	18 (66.7)	10 (66.7)	2 (18.2)
Pediatric surgeon	18 (66.7)	12 (80.0)	7 (63.6)
General surgeon	4 (14.8)	1 (6.7)	NA
ENT surgeon	13 (48.1)	8 (53.3)	2 (18.2)
Geneticist	4 (14.8)	1 (6.7)	NA
PHO	2 (7.4)	NA	1 (9.1)
Other	9 (33.3)	2 (13.3)	4 (36.4)

Abbreviations: ENT, ear, nose, and throat; NA, not applicable; PHO, pediatric hematologist-oncologist; VAC, vascular anomaly center.

Peripheral blood samples for genetic testing were commonly sent to Invitae (27 of 53 [50.9%]), Seattle Children’s Hospital Molecular Genetics Laboratory (16 of 53 [30.2%]), Washington University in St Louis NGS Laboratory (15 of 53 [28.3%]), in-house (respondent’s institution) clinical testing (15 of 53 [28.3%]), and Foundation Medicine (14 of 53 [26.4%]) (Figure). For genetic testing on tissue, Washington University in St Louis NGS Laboratory (19 of 53 [35.8%]) was the laboratory most frequently cited, followed by Seattle Children’s Hospital Molecular Genetics Laboratory (17 of 53 [32.1%]), in-house clinical testing (17 of 53 [32.1%]), and Invitae (16 of 53 [30.2%]). In-house clinical testing was more common at large and midsize centers. In-house research testing for blood sample was reported at only 2 of 27 large VACs (7.4%), and in-house research testing for tissue sample was reported at 4 of 27 large VACs (14.8%). Small VACs were more likely to use Tempus and Foundation Medicine for testing, which are platforms that were designed for genetic testing of cancers.

**Figure.**
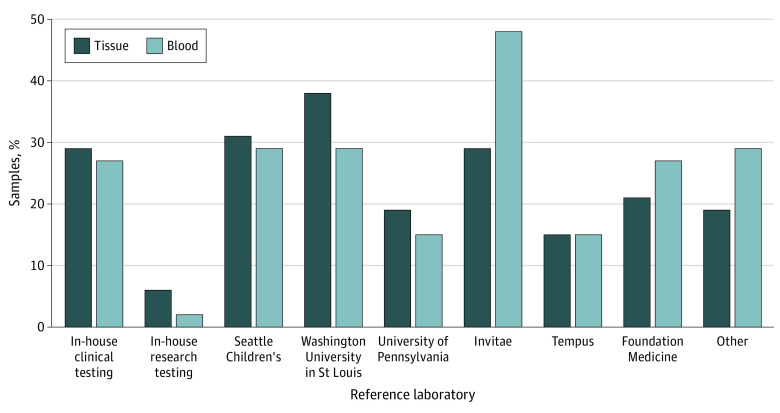
Reference Laboratories for Genetic Testing in Vascular Malformations Dark blue bars represent where a tissue specimen was sent for somatic testing, and light blue bars represent where peripheral blood samples were sent for germline testing. Other less frequently used laboratories included Prevention Genetics, GeneDx, ARUP, Ambry Genetics, Mayo Medical Laboratories, Blueprint Genetics, Caris, and Cincinnati Children’s Molecular Genetics Laboratory.

At 36 of 53 VACs (67.9%), the person ordering the test decided which laboratory to use for testing blood and tissue specimens. Respondents who selected the other or combination option for these questions commented that the decision was shared between the ordering clinical physician and a geneticist or genetics counselor, with influence by insurance coverage. The institution dictated which laboratory was to be used for genetic testing at 9 of 53 VACs (17.0%). Paired testing with both blood and tissue samples was common (23 of 53 respondents [43.4%]) but varied with clinical diagnosis, requirements of the laboratory, or insurance. Most respondents (28 of 53 [52.8%]) reported needing to place separate orders for blood and tissue specimens. Among 53 respondents, biopsies for tissue testing were commonly performed by pediatric surgeons (37 [69.8%]); dermatologists (33 [62.3%]); interventional radiologists (30 [56.6%]); ear, nose, and throat surgeons (23 [43.4%]); and PHOs (3 [5.7%]). The other category included plastic and orthopedic surgeons.

Most respondents (30 of 53 [56.6%]) requested a gene panel when ordering genetic testing. Others (17 of 53 [32.1%]), with concerns that insurance would not cover a somatic overgrowth panel, ordered sequencing of a single gene, with reflex to broader testing if the initial testing results were negative.

### Insurance Issues

Processes for insurance approval for genetic testing are summarized in Table 3. Prior authorization for genetic testing was commonly obtained by an administrator in the VAC (17 of 53 respondents [32.1%]), an insurance coordinator (13 [24.5%]), a registered nurse (13 [24.5%]), or a PHO (12 [22.6%]). However, the appeals process following an insurance denial was primarily done by the ordering PHO (35 of 53 VACs [66.0%]). Those who selected the other category for approvals and denials listed a geneticist or genetics counselor. Only large VACs reported that the institutional laboratory obtained authorization. Several large and midsize centers reported having genetic counselors available for the authorization and appeals processes, but no small centers had this type of support.

**Table 3.** Insurance Issues Reported for Genetic Testing by 53 Respondents

Survey question	Respondents, No. (%)		
	Large VAC (n = 27)	Midsize VAC (n = 15)	Small VAC (n = 11)
Who obtains prior authorization for genetic testing?			
Insurance coordinator	7 (25.9)	4 (26.7)	2 (18.2)
RN	5 (18.5)	6 (40.0)	2 (18.2)
Administrator	6 (22.2)	7 (46.7)	4 (36.4)
PHO	7 (25.9)	4 (26.7)	1 (9.1)
Patient	4 (14.8)	NA	NA
Institutional laboratory	7 (25.9)	1 (6.7)	NA
Referral laboratory	6 (22.2)	1 (6.7)	NA
I don’t know	1 (3.7)	1 (6.7)	4 (36.4)
Other	8 (29.6)	2 (13.3)	NA
If there is insurance denial, who does the appeal?			
Insurance coordinator	3 (11.1)	NA	1 (9.1)
RN	8 (29.6)	2 (13.3)	2 (18.2)
Administrator	5 (18.5)	1 (6.7)	NA
PHO	17 (63.0)	12 (80.0)	6 (54.5)
Patient	2 (7.4)	NA	NA
Institutional laboratory	2 (7.4)	NA	NA
Referral laboratory	1 (3.7)	NA	NA
I don’t know	2 (7.4)	1 (6.7)	3 (27.2)
Other	10 (37.0)	1 (6.7)	NA
Who discusses the finances of genetic testing with the patient/family?			
Ordering physician or nurse	19 (70.4)	10 (66.7)	7 (63.6)
Ordering genetic counselor	14 (51.9)	10 (66.7)	2 (18.2)
Ordering administrator	2 (7.4)	NA	NA
Financial counselor	2 (7.4)	2 (13.3)	NA
Social worker	1 (3.7)	1 (6.7)	1 (9.1)
Reference/genetic testing laboratory	5 (18.5)	1 (6.7)	NA
Other	1 (3.7)	NA	NA
I don’t know	2 (7.4)	1 (6.7)	3 (27.3)
What insurance carriers does your group mostly interact with?			
State Medicaid	26 (96.3)	14 (93.3)	10 (90.9)
Medicare	4 (14.8)	2 (13.3)	1 (9.1)
BCBS	19 (70.4)	11 (73.3)	7 (63.6)
Aetna	15 (55.6)	5 (33.3)	4 (36.4)
Tricare	11 (40.7)	6 (40.0)	2 (18.2)
United Health	19 (70.4)	5 (33.3)	5 (45.5)
Anthem	10 (37.0)	6 (40.0)	NA
Humana	5 (18.5)	2 (13.3)	1 (9.1)
Kaiser	3 (11.1)	NA	NA
Self-pay	5 (18.5)	1 (6.7)	NA
Other	7 (25.9)	2 (13.3)	2 (18.2)

Abbreviations: BCBS, Blue Cross Blue Shield; NA, not applicable; PHO, pediatric hematologist-oncologist; RN, registered nurse; VAC, vascular anomaly center.

Many respondents (19 of 53 [35.8%]) reported needing a follow-up insurance authorization if a panel was requested after failure of diagnostic single-gene testing. One-third of respondents (17 of 53 [32.1%]) reported that insurance approval was obtained between 50% and 74% of the time on the first attempt. One-quarter of respondents (13 of 53 [24.5%]) reported a higher rate of approval—between 75% and 99%—on the first attempt. Twelve of 53 respondents (22.6%) reported not knowing how often approval was obtained.

Despite most respondents commenting that a discussion with patients and families about insurance approval and testing costs was important, not all were sure that this conversation was happening in their clinics. Only 33 of 53 respondents (62.3%) could confirm that a conversation about financing genetic testing was occurring with patients and families. Only 1 of 53 respondents (1.9%) from a large VAC reported that an institutional managed care coordinator held these conversations. Three of 53 respondents (5.7%) reported such conversations did not happen at all. Three respondents (5.7%) reported that in 3 VACs no conversations about financing were occurring prior to genetic testing. In 6 of 53 VACs (11.3%), the respondents stated they did not know who was responsible for discussing the financial implications of genetic testing with patients. Most of the patient and family conversations about the financing of genetic testing appeared to be led by the ordering physician or nurse (36 of 53 VACs [67.9%]) or a genetic counselor (26 [49.1%]). Several responses suggested that some families may not be receiving any financial guidance prior to undergoing testing. Five of 53 respondents (9.4%) cited difficulty finding accurate information about insurance approval and testing costs prior to testing. Respondents also reported interacting with many different insurance carriers, and nearly all respondents (50 of 53 [94.3%]) reported working with state Medicaid entities. Health insurance carriers that were grouped under the other category included state-specific plans, Cigna, and a few smaller carriers.

### Perceived Effort of Genetic Testing

Multiple respondents described their efforts to obtain genetic testing as “an uphill battle,” “monumental effort,” or “huge burden of time.” Many respondents commented on needing to spend multiple hours per patient on the process, and several admitted that their own lack of knowledge about the system and process was a barrier. While a few respondents reported adequate support for the processes surrounding genetic testing on patients with vascular anomalies, most reported that support was insufficient. A few VACs, even those with good support, experienced delays in obtaining insurance approval or dealing with denials. Issues with genetic testing specifically associated with VM included lack of familiarity with the diagnosis codes used by insurance companies and lack of precise diagnostic coding for vascular anomalies. Many respondents noted that the entire process of obtaining genetic testing for patients with cancer was far easier than for patients with vascular anomalies.

### Finance-Related Differences Between Laboratories

As shown in Table 4, billing procedures and cost to patients varied among and within laboratories, depending on whether a patient had private or public insurance. Of the 4 most commonly used laboratories, only Invitae and Seattle Children’s Hospital Molecular Genetics Laboratory initiated benefits investigations and directly contacted families with private insurance about their financial obligations. Invitae also offered a capitated maximum obligation of $250 for any single order regardless of insurance coverage, and Seattle Children’s Hospital Molecular Genetics Laboratory offered a financial assistance program. Some laboratories billed institutions directly, unless the patient was designated as self-pay at the time the test was ordered. Washington University in St Louis NGS Laboratory provided benefits investigations when insurance was billed directly. Two laboratories did not add charges when single-gene testing reflexed to more comprehensive panels. The Seattle Children’s Hospital Molecular Genetics Laboratory and the Washington University in St Louis NGS Laboratory offered Medicaid billing in-state only. Some institutions contracted with Invitae to offer direct Medicaid billing.

**Table 4.** Comparison of Billing Procedures and Costs at Reference Laboratories

Procedure	Invitae	University of Pennsylvania Genetic Diagnostic Laboratory	Seattle Children’s Hospital Molecular Genetics Laboratory	Washington University in St Louis NGS Laboratory
Initiates benefits investigation?	Yes	No	Yes	No
Contacts patient about out-of-pocket expenses?	Yes	Bills institution or patient as self-pay	Yes	Bills Aetna, Anthem, Cigna, Missouri Medicaid or Medicare, United Healthcare, institution or patient as self-pay
Options for patients	Out-of-pocket costs or pay option of $250	Self-pay or submit receipt to insurance company	Offers discussion with financial counselor and financial assistance for self-pay (including installment program and 15% discount)	Self-pay or submit receipt to insurance company
Options for reflex from single gene tests to gene panels	Reflex to an additional panel from the same sample has no additional cost if performed on the same sample within 150 d of original report date	Gene panel only, except for HHT (can perform single-gene sequencing)	No, but offers more targeted panels for specific VMs	Subset panels are available to be reflexed for no additional cost to their full 37-gene platform
Patient assistance program	Offered for patients with high copay	No	Yes	NA
Process variation for private insurer vs Medicaid or Medicare?	No billing or balance billing to patient; primarily does institutional billing, but some institutions contract with Invitae for direct Medicaid billing	Offers institutional billing	Cannot bill out-of-state Medicaid (except Alaska, Idaho, and Montana) and cannot bill Medicare unless an established Seattle Children’s patient	Bills sent to Missouri Medicaid or Medicare; all other bills sent to institution or patient as self-pay

Abbreviations: HHT, hereditary hemorrhagic telangiectasia; NA, not applicable; VM, vascular malformation.

## Discussion

The overall prevalence of VMs in the US has been estimated at 1.5% of the general population.^14^ Disproportionate to their prevalence, many patients with VM experience substantial morbidities that interfere with their quality of life, including functional impairment, chronic pain, infection, and increased mortality. These patients require a comprehensive approach to care that includes physicians from multiple specialties, diagnostic imaging, histopathology, access to surgery and interventional procedures, and molecular diagnostic measures to support targeted medical therapy.^2,10,15,16^

Medical management of VM has shifted from symptom care alone to targeted therapies focused on controlling the genetic drivers of these diseases.^1,17^ The introduction of sirolimus for the management of VM represented a paradigm shift in the approach to medical management.^7,8^ While sirolimus remains a critical part of the therapeutic armamentarium, data increasingly suggest that more precise targeting of gene variants may result in better outcomes.^18,19,20,21,22,23^ Because many of the genetic drivers of VM are shared with those found in cancers, most drugs under investigation have been borrowed from cancer treatments. Alpelisib (PIQRAY and Vijoice; Novartis), a drug that targets the *PIK3CA* gene, was approved in April 2022 by the US Food and Drug Administration for the treatment of a specific group of VM disorders: *PIK3CA*-Related Overgrowth Spectrum.^22,23,24^ Other drugs, such as trametinib (Mekinist; Novartis) and cobimetinib, which target MEK, and miransertib, which targets AKT, are in clinical trials or are in early development.^1,25,26,27,28,29,30^

Despite growing evidence that targeted therapies are an important component of comprehensive care for patients with VM, the results of the present survey underscore the difficulties in accessing genetic testing for this group of disorders. Respondents, most of whom were PHOs, universally noted that the obstacles in getting genetic information were unique to VM and were not similar to issues they often faced in obtaining molecular testing for patients with cancer. Most respondents also noted seeing a substantial increase in genetic testing for VM and thus anticipating further increases. Because genetic testing for VM is rapidly becoming standard of care, it is critical to address barriers to testing.

Highlighted concerns included lack of administrative support for ordering genetic testing, difficulties in obtaining insurance coverage, and specific limitations of genetic testing in this population (ie, low levels of variant allele expression in tissue). Insurance denials, multiple orders (separate for blood and tissue testing), additional approvals for second-line testing, and appeals with requirements for peer-to-peer interactions were universal issues. We also confirmed considerable variation in insurance coverage among laboratories.

Many of the difficulties in obtaining genetic testing for VM were magnified at small and midsize VACs. In-house genetic testing was much more available at large and midsize centers, and in-house research testing was observed only at large centers. Small VACs sent a higher proportion of testing to Foundation Medicine and Tempus, which are genomic profiling companies designed for oncology. Small centers also reported fewer resources for genetic testing, including fewer team members available for administrative processes, less availability of clinical geneticists or genetic counselors, and more uncertainty about testing processes. Clinicians from small centers were also less likely to be a part of national consortia for vascular anomalies (eg, CaNVAS) and less likely to have true multidisciplinary teams. The results confirmed findings from an unpublished study by the PHO VA Interest Group on VAC resources across the US (S. Cohen-Cutler, MD, MS, personal communication, January 20, 2023), which noted that relatively few centers care for this population. Presumably, many patients are not tested and therefore may not benefit from emerging drug treatment options.

The method of genetic testing for VM is critical, as most are due to somatic, postzygotic sequence variations and will not be identified using standard methods for germline conditions. While the focus of this survey was on somatic disorders, some respondents included notes about testing for germline disorders. Notably, VACs of all sizes sent specimens (including tissue) to Invitae, a laboratory that is not validated for variant allele frequencies less than 20%. Such sequencing methods, although adequate for cancer evaluation, may miss detection of sequence variations at the low variant allele frequencies seen in many VMs.^11,30,31^ Standard sequencing techniques for oncological diagnosis can fail to identify low-frequency somatic variants in VM, which can result in missed identification of relevant variants.^31,32,33,34^ Of note, there were differences in the ordering practices for blood vs tissue specimens in this survey, possibly due to involvement of different clinicians involved with biopsy or specimen processing.

A major theme from this survey was that many respondents believed they lacked knowledge about the process of genetic testing for VM. Most respondents were PHOs, who self-reported that they were one of the major groups responsible for ordering genetic testing for VM. Unlike geneticists and genetic counselors, PHOs receive little formal training in genetic testing methods and genetic counseling. Many respondents expressed that their uncertainty about their knowledge of genetic testing was a major barrier. This opinion was particularly striking in the questions about providing counseling prior to genetic testing, with less than half of the respondents reporting that conversations with a genetic counselor were occurring prior to testing and several respondents reporting that no such discussions were occurring. It is important for all patients and families to be counseled appropriately prior to genetic testing, and it is clear from this survey that this step is lacking. Ideally, geneticists and genetic counselors would be part of every multidisciplinary team for VM, but for VACs without these resources, additional educational support for clinicians who order genetic testing might be worthwhile.

### Guidance for Supporting Genetic Testing

A small number of respondents noted that their institutions recognized the downstream benefits of genetic testing for vascular anomalies, both in providing appropriate care for this patient population and in generating revenue and cost savings. While genetic testing is expensive, the benefit to the institution of billing for the procedure for biopsy and ongoing patient care once a patient has started targeted medical therapy should not be overlooked. Financial details were not examined in this survey.

We believe a change in approach to genetic testing across the national PHO VA Interest Group could be implemented to yield substantial benefit. This approach could include educational modules and logistical support for physicians caring for patients with VMs. An itemization of the *International Statistical Classification of Diseases and Related Health Problems, Tenth Revision* codes relevant to vascular anomalies was developed by the PHO VA Interest Group and could be linked to laboratory ordering forms and made more generally available. The Cleveland Clinic has previously reported substantial cost savings for genetic testing from implementing a restricted-use initiative that limits ordering of genetic tests to those clinicians who routinely do such ordering.^35^ While restricted use may not be feasible at small VACs, one can envision a role for a more centralized support or mentoring team to provide education and support to small centers. Currently, there is a national web-based forum through which PHOs can seek input from a rotating group of national experts. Updated educational and logistical materials on genetic testing for VM could be maintained centrally, including key aspects of counseling, important considerations for sequencing methods, diagnostic and billing procedure codes, and templates of insurance authorization letters. Educational sessions on genetic testing for VM could be incorporated into conferences targeted at PHOs treating vascular anomalies. Although not a replacement for dedicated personnel with genetic testing training, access to experts could serve as a stopgap measure for clinicians lacking such support. Ultimately, more widespread access to vascular anomalies experts, including geneticists and genetic counselors, would facilitate testing. The findings of this survey reflect barriers to genetic testing in the US, which has both unique benefits and inadequacies compared with systems in European and other countries.^36,37,38,39^

### Limitations

There were several limitations to this study, including the recall bias inherent in most cross-sectional surveys. The surveys were completed primarily by 1 PHO from each institution, rather than vascular anomalists from other specialties at that institution, potentially leading to bias. In particular, the perspective of geneticists and genetic counselors who see and test these patients outside of a multidisciplinary setting may be missing. However, respondents were asked to solicit input from other team members to better reflect the practices for testing patients with VM at their institution. While the emphasis of this survey was on somatic testing for VM, some respondents included notes about the testing they performed for germline conditions. Although these comments were easily abstractable from the responses, the differences in responses could have affected the results. The focus of this study was on the logistics and effort of genetic testing in patients with VM, not on obtaining targeted medications based on genetic testing results. Although separate, these issues are tightly linked due to the need for confirmatory genetic testing to obtain drug approval.

## Conclusions

In this survey study, we identified barriers to genetic testing for VM across VACs, described differences between VACs based on size, and proposed multiple interventions to support clinicians ordering genetic testing for VM. Because genetic testing for VM is rapidly becoming the standard of care, it is critical to identify and address the barriers to such testing. Barriers to genetic testing in VM are likely to be similar in other rare diseases. The results and recommendations of this study should have broader application not only to clinicians caring for patients for whom molecular diagnosis is important to medical management but also to health care and genomic medicine.

## Notes

Supplement 1. **eAppendix.** REDCap Survey

Supplement 2.
Data Sharing Statement
