# Supplementary material for: Barriers to Genetic Testing in Vascular Malformations
Source: JAMA Netw Open. 2023 May 23;6(5):e2314829. doi: 10.1001/jamanetworkopen.2023.14829 (PMC10208144; doi:10.1001/jamanetworkopen.2023.14829)
Supplement: Supplement 1. — eAppendix. REDCap Survey [file jamanetwopen-e2314829-s001.pdf]

Supplemental Online Content

Borst AJ, Hammill AM, Crary SE, McLean TW, Felton T, Blatt J. Barriers to genetic testing in vascular malformations. *JAMA Netw Open*. 2023;6(5):e2314829. doi:10.1001/jamanetworkopen.2023.14829

eAppendix. REDCap Survey

This supplemental material has been provided by the authors to give readers additional information about their work.

© 2023 Borst AJ et al. *JAMA Network Open*.

Hello Colleagues,

You are receiving this survey as a member of the ASPHO Vascular Anomalies SIG. The goal of this survey is to obtain more information about how each center or ASPHO VA SIG member is approaching genetic testing for patients with vascular anomalies. This includes potential pitfalls with regards to insurance authorization and denials. We are hopeful that this survey will provide us with some information to publish and disseminate and hopefully improve these processes for all of us in the future.

We are guessing you are sick of surveys and will sweeten the ask by offering a $5 Dunkin Donuts gift card that expires 6/21/22. Having said that, you may not have all the answers to our questions and may want to look at the PDF of the survey attached in advance of completing the survey by the link. We pulled one person per institution and if you think someone else would be more suited to this task, please pass it along. We are asking for your name and institution in the survey so that we can contact you with any questions and also give you credit if we publish.
However, you do not need to provide this information in order to participate. We think that the information we get (and publish) may nudge institutions to get more support for this nuisance aspect of our job.

There are 3 pages and 33 questions in total. We estimate no longer than 15 minutes to complete. Thank you so much for participating!

---

1 What is your name and title?

____

2 What is the name of your institution?

____

What is your email address if we need to contact you?

____

3 Do you work for a public or private institution?

- ○ Public
- ○ Private
- ○ Other

3a What other type of institution do you work for?

____

4 Do you have a specific clinic or program dedicated to vascular anomalies at your institution?

- ○ Yes
- ○ No
- ○ Sort of
- ○ I'm not sure

4a Any additional information to add about vascular anomalies clinic at your institution?

____

© 2023 Borst AJ et al. *JAMA Network Open*.

5	What is your role within the vascular anomalies clinic or program at your center? Check all that apply.	☐ Physician (heme-onc)<br>☐ Physician (other)<br>☐ Nurse<br>☐ Nurse Practitioner<br>☐ Geneticist<br>☐ Genetic counselor<br>☐ Research assistant<br>☐ Administrator<br>☐ Director<br>☐ Co-Director<br>☐ Member<br>☐ Other
5a	What is your other role?	__________________________________
6	Are you a current member of the ASPHO Vascular Anomalies Special Interest Group?	◯ Yes<br>◯ No
7	Are you a current member of CaNVAS (Consortium of iNvestigators of Vascular AnomalieS)?	◯ Yes<br>◯ No
8	Are you a current member of ISSVA (International Society for the Study of Vascular Anomalies)?	◯ Yes<br>◯ No
9	How many patients per year with vascular malformations do you estimate are seen by your vascular anomalies clinic/center?	◯ > 100 unique patients per year<br>◯ 25-100 unique patients per year<br>◯ < 25 unique patients per year<br>◯ I'm not sure
10	What is the maximum age of patients you see in your clinic? Please write n/a or no max age if one does not exist.	____________________________

© 2023 Borst AJ et al. *JAMA Network Open*.

Information about ordering genetic testing

Hello Colleagues,

You are receiving this survey as a member of the ASPHO Vascular Anomalies SIG. The goal of this survey is to obtain more information about how each center or ASPHO VA SIG member is approaching genetic testing for patients with vascular anomalies. This includes potential pitfalls with regards to insurance authorization and denials. We are hopeful that this survey will provide us with some information to publish and disseminate and hopefully improve these processes for all of us in the future.

We are guessing you are sick of surveys and will sweeten the ask by offering a $5 Dunkin Donuts gift card that expires 6/21/22. Having said that, you may not have all the answers to our questions and may want to look at the PDF of the survey attached in advance of completing the survey by the link. We pulled one person per institution and if you think someone else would be more suited to this task, please pass it along. We are asking for your name and institution in the survey so that we can contact you with any questions and also give you credit if we publish. However, you do not need to provide this information in order to participate. We think that the information we get (and publish) may nudge institutions to get more support for this nuisance aspect of our job.

There are 3 pages and 33 questions in total. We estimate no longer than 15 minutes to complete.

Thank you so much for participating!

---

11 Approximately how many patients with vascular anomalies get genetic testing at your institution each year?

You can feel free to confirm with your genetic testing/referral laboratory or provide your best guess estimate.

- ◯ < 5
- ◯ 5-10
- ◯ 10-20
- ◯ 20-50
- ◯ 50-100
- ◯ 100-200
- ◯ > 200
- ◯ I do not know

---

12 Has the number of genetic tests sent on patients with vascular anomalies gone up or down over the past 3 years?

- ◯ Has gone way up (> 10 times more tests)
- ◯ Has gone up (> 2-5 times more tests)
- ◯ Is about the same
- ◯ Has gone down (by 1/3 or less)
- ◯ Has gone way down (by > 1/3 or more)
- ◯ I do not know

---

13 What percentage of genetic testing in patients with vascular anomalies at your institution is for pediatric patients age < 18 years?

- ◯ > 90%
- ◯ 50% or more
- ◯ 25-50%
- ◯ < 25%
- ◯ None
- ◯ I do not know

---

14 Who places orders for genetic testing in vascular anomalies patients at your institutions (check all that apply)?

- ☐ Geneticist
- ☐ Genetic counselor
- ☐ Hematologist-oncologist
- ☐ Nurse
- ☐ Nurse practitioner
- ☐ Research assistant
- ☐ Other administrative staff (can specify more detail)
- ☐ Another physician (can specify more detail)

14a Specify other administrative staff or other type of physician who orders genetic testing

____

© 2023 Borst AJ et al. *JAMA Network Open*.

15 Any comments on who orders genetic testing at your institution?

____

14b Approximately how many geneticists are ordering genetic testing on patients with vascular anomalies at your institution?

- ◯ 1
- ◯ 2
- ◯ 3
- ◯ 4
- ◯ 5
- ◯ More than 5
- ◯ I do not know
- ◯ None

14c Approximately how many genetic counselors are ordering genetic testing on patients with vascular anomalies at your institution?

- ◯ 1
- ◯ 2
- ◯ 3
- ◯ 4
- ◯ 5
- ◯ More than 5
- ◯ I do not know
- ◯ None

14d Approximately how many hematologists and/or oncologists are ordering genetic testing on patients with vascular anomalies at your institution?

- ◯ 1
- ◯ 2
- ◯ 3
- ◯ 4
- ◯ 5
- ◯ More than 5
- ◯ I do not know
- ◯ None

14e Approximately how many nurses are ordering genetic testing on patients with vascular anomalies at your institution?

- ◯ 1
- ◯ 2
- ◯ 3
- ◯ 4
- ◯ 5
- ◯ More than 5
- ◯ I do not know
- ◯ None

14f Approximately how many nurse practitioners are ordering genetic testing on patients with vascular anomalies at your institution?

- ◯ 1
- ◯ 2
- ◯ 3
- ◯ 4
- ◯ 5
- ◯ More than 5
- ◯ I do not know
- ◯ None

14g Approximately how many research assistants are ordering genetic testing on patients with vascular anomalies at your institution?

- ◯ 1
- ◯ 2
- ◯ 3
- ◯ 4
- ◯ 5
- ◯ More than 5
- ◯ I do not know
- ◯ None

© 2023 Borst AJ et al. *JAMA Network Open*.

14h Approximately how many other administrative staff are ordering genetic testing on patients with vascular anomalies at your institution?

- ◯ 1
- ◯ 2
- ◯ 3
- ◯ 4
- ◯ 5
- ◯ More than 5
- ◯ I do not know
- ◯ None

14i Approximately how many other physicians are ordering genetic testing on patients with vascular anomalies at your institution?

- ◯ 1
- ◯ 2
- ◯ 3
- ◯ 4
- ◯ 5
- ◯ More than 5
- ◯ I do not know
- ◯ None

16 Where do you send genetic testing from BLOOD on patients with vascular anomalies? Check all that apply

- ☐ Our own institution (in-house CLINICAL testing)
- ☐ Our own institution (in-house RESEARCH testing)
- ☐ University of Washington, Seattle, WA
- ☐ Washington University in St. Louis
- ☐ University of Pennsylvania
- ☐ Invitae
- ☐ Tempus
- ☐ Foundation Medicine
- ☐ Other

16a Where else do you send genetic testing from BLOOD on patients with vascular anomalies?

____

17 Who decides what laboratory to send BLOOD testing to?

- ☐ Insurance
- ☐ Person ordering the test
- ☐ Institution
- ☐ Genetic counselor
- ☐ Other or combination

17a Who else decides where to send BLOOD testing or is it a combined decision?

____

18 Where do you send genetic testing from the TISSUE on patients with vascular anomalies? Check all that apply

- ☐ Our own institution (in-house CLINICAL testing)
- ☐ Our own institution (in-house RESEARCH testing)
- ☐ University of Washington, Seattle, WA
- ☐ Washington University in St. Louis
- ☐ University of Pennsylvania, Perelman Laboratory
- ☐ Invitae
- ☐ Tempus
- ☐ Foundation Medicine
- ☐ Other

18a Where else do you send genetic testing from TISSUE on patients with vascular anomalies?

____

© 2023 Borst AJ et al. *JAMA Network Open*.

19 Who decides what laboratory to send TISSUE testing to?

- ☐ Insurance
- ☐ Person ordering the test
- ☐ Institution
- ☐ Genetic counselor
- ☐ Other or combination

19a Who else decides where to send TISSUE testing or is it a combined decision?

____

20 Do genetic testing samples for BLOOD and TISSUE go to the same laboratory?

- ◯ Yes
- ◯ No
- ◯ It depends
- ◯ I do not know

20a What does it depend on?

____

21 Are separate orders required for paired blood and tissue samples?

- ◯ Yes
- ◯ No
- ◯ It depends
- ◯ I do not know

21a What does it depend on?

____

22 Do you usually order a single gene analysis or gene panel?

- ☐ Single gene only
- ☐ Single gene with reflex to gene panel if negative
- ☐ Gene panel
- ☐ It depends on clinical scenario

22a What does it depend on?

____

23 If a single gene assay is done and then a panel is desire, are additional orders required?

- ◯ Yes
- ◯ No
- ◯ It depends
- ◯ I do not know

23a What does it depend on?

____

24 Who performs the biopsy to obtain tissue specimen for genetic testing for patients with vascular anomalies (check all that apply)?

- ☐ Dermatology
- ☐ Interventional Radiology
- ☐ Pediatric Surgery
- ☐ General Surgery
- ☐ ENT
- ☐ Genetics
- ☐ Pediatric Hematology-Oncology
- ☐ Other

24a Who else performs biopsy for tissue specimen for genetic testing?

____

© 2023 Borst AJ et al. *JAMA Network Open*.

Insurance issues

Hello Colleagues,

You are receiving this survey as a member of the ASPHO Vascular Anomalies SIG. The goal of this survey is to obtain more information about how each center or ASPHO VA SIG member is approaching genetic testing for patients with vascular anomalies. This includes potential pitfalls with regards to insurance authorization and denials. We are hopeful that this survey will provide us with some information to publish and disseminate and hopefully improve these processes for all of us in the future.

We are guessing you are sick of surveys and will sweeten the ask by offering a $5 Dunkin Donuts gift card that expires 6/21/22. Having said that, you may not have all the answers to our questions and may want to look at the PDF of the survey attached in advance of completing the survey by the link. We pulled one person per institution and if you think someone else would be more suited to this task, please pass it along. We are asking for your name and institution in the survey so that we can contact you with any questions and also give you credit if we publish. However, you do not need to provide this information in order to participate. We think that the information we get (and publish) may nudge institutions to get more support for this nuisance aspect of our job.

There are 3 pages and 33 questions in total. We estimate no longer than 15 minutes to complete.

Thank you so much for participating!

---

25 Who obtains prior authorization from insurance for genetic testing for vascular anomalies patients? Choose all that apply.

- ☐ Insurance coordinator
- ☐ RN calls insurance
- ☐ Someone in our administrative staff calls insurance
- ☐ I call insurance
- ☐ The patient has to call their own insurance
- ☐ Our institutional lab calls the insurance
- ☐ The referral lab calls the insurance
- ☐ I don't know
- ☐ Other

25a Who else obtains insurance authorization for genetic testing for patients with vascular anomalies?

____

---

26 If there is an insurance denial, who does the appeal for testing?

- ☐ Our insurance coordinator does this
- ☐ Our RN does this
- ☐ Someone in our administrative staff does this
- ☐ I do this
- ☐ The patient has to do this
- ☐ Our institution does this
- ☐ The referral lab does this
- ☐ I don't know
- ☐ No one helps with this
- ☐ Other

26a Who else helps with appeals after insurance denial?

____

---

27 If a single gene assay is done and then a panel is desire, is new insurance authorization required?

- ○ Yes
- ○ No
- ○ It depends
- ○ I do not know

27a What does it depend on?

____

© 2023 Borst AJ et al. *JAMA Network Open*.

28 Approximately how often is approval obtained after the first attempt?

- ◯ 100% of the time
- ◯ 75-99% of the time
- ◯ 50-74% of the time
- ◯ 25-49% of the time
- ◯ < 25% of the time
- ◯ I do not know

29 Are patients/families told about the finances of genetic testing prior to sending testing?

- ◯ Yes
- ◯ No
- ◯ I think this happens most of the time
- ◯ I do not know

Any comments about discussing finances of genetic testing with patients/families?

____

30 Who discusses the finances of genetic testing with the family?

- ☐ Ordering physician or nurse
- ☐ Ordering genetic counselor
- ☐ Ordering administrative person
- ☐ Financial counselor
- ☐ Social worker
- ☐ Reference/genetic testing lab
- ☐ Other
- ☐ I do not know

30a Who else discusses the finances of genetic testing with the family?

____

31 What insurance carriers does your group mostly interact with? Check all that apply

- ☐ State Medicaid
- ☐ Medicare
- ☐ BCBS
- ☐ Aetna
- ☐ Tricare
- ☐ United Health
- ☐ Anthem
- ☐ Humana
- ☐ Kaiser
- ☐ Self-pay
- ☐ Other

31a What other insurers does your group interact with?

____

32 How much of an effort do you feel that it is to get genetic testing for this group of patients? Do you feel that you have adequate support for this effort at your institution? If not, what do you need and what would you like to see in terms of additional support?

____

33 If you also follow patients with malignancies, please feel free to compare you effort getting genetic testing and tissue from this patient population vs those with malignancy.

____

© 2023 Borst AJ et al. *JAMA Network Open*.
