# Supplementary material for: Barriers to Genetic Testing in Vascular Malformations
Source: JAMA Netw Open. 2023 May 23;6(5):e2314829. doi: 10.1001/jamanetworkopen.2023.14829 (PMC10208144; doi:10.1001/jamanetworkopen.2023.14829)
Supplement: Supplement 2. — Data Sharing Statement [file jamanetwopen-e2314829-s002.pdf]

Data Sharing Statement

Borst. Barriers to Genetic Testing in Vascular Malformations. *JAMA Netw Open*. Published May 23, 2023. doi:10.1001/jamanetworkopen.2023.14829

Data
Data available: Yes
Data types: Deidentified participant data
How to access data: Individual request to borsta@chop.edu
When available: With publication

Supporting Documents
Document types: Other (please specify)
Additional Information: survey data
How to access documents: email request to borsta@chop.edu
When available: With publication

Additional Information
Who can access the data: to anyone requesting the data
Types of analyses: survey information
Mechanisms of data availability: email request to borsta@chop.edu
